# Supplementation With 2′-FL and scGOS/lcFOS Ameliorates Rotavirus-Induced Diarrhea in Suckling Rats

**DOI:** 10.3389/fcimb.2018.00372

**Published:** 2018-10-23

**Authors:** Ignasi Azagra-Boronat, Malén Massot-Cladera, Karen Knipping, Belinda van't Land, Bernd Stahl, Johan Garssen, Maria José Rodríguez-Lagunas, Àngels Franch, Margarida Castell, Francisco J. Pérez-Cano

**Affiliations:** ^1^Physiology Section, Department of Biochemistry and Physiology, Faculty of Pharmacy and Food Science, University of Barcelona, Barcelona, Spain; ^2^Nutrition and Food Safety Research Institute (INSA-UB), Barcelona, Spain; ^3^Nutricia Research, Utrecht, Netherlands; ^4^Division of Pharmacology, Faculty of Science, Utrecht Institute for Pharmaceutical Sciences, Utrecht University, Utrecht, Netherlands; ^5^Department of Pediatric Immunology, University Medical Centre Utrecht/Wilhelmina Children's Hospital, Utrecht, Netherlands

**Keywords:** 2′-FL, scGOS/lcFOS, prebiotic, rotavirus, diarrhea, suckling rats

## Abstract

Rotavirus (RV) is considered to be the most common cause of gastroenteritis among infants aged less than 5 years old. Human milk bioactive compounds have the ability to modulate the diarrheic process caused by several intestinal pathogens. This study aimed to evaluate the potential protective role of a specific human milk oligosaccharide, 2′-fucosyllactose (2′-FL), a mixture of the prebiotic short-chain galacto-oligosaccharides and long-chain fructo-oligosaccharides 9:1 (GOS/FOS) and their combination (2′-FL+GOS/FOS) on RV-induced diarrhea in suckling rats. The nutritional intervention was performed from the second to the sixteenth day of life by oral gavage and on day 5 an RV strain was orally administered to induce infection. Fecal samples were scored daily to assess the clinical pattern of severity, incidence and duration of diarrhea. Blood and tissues were obtained at day 8 and 16 in order to evaluate the effects on the epithelial barrier and the mucosal and systemic immune responses. In the assessment of severity, incidence and duration of diarrhea, both 2′-FL and GOS/FOS displayed a beneficial effect in terms of amelioration. However, the mechanisms involved seemed to differ: 2′-FL displayed a direct ability to promote intestinal maturation and to enhance neonatal immune responses, while GOS/FOS induced an intestinal trophic effect and an RV-blocking action. The combination of 2′-FL and GOS/FOS showed additive effects in some variables. Therefore, it could be a good strategy to add these compounds in combination to infant formulas, to protect against human RV-induced diarrhea in children.

## Introduction

Human milk provides several bioactive factors that benefit the relatively immature immune system of neonates early in life. These components have been categorized into two different groups according to either their protective role or their ability to promote maturation (Lewis et al., 2017). In this sense, human milk oligosaccharides (HMOs) are considered to play a part both in protection and maturation.

Most of the 200 types of HMOs discovered exhibit a lactose, polylactosamine or lacto-N-biose core, which is bound to either fucose or sialic acids. This structure confers on them a high chemical variability and protection from digestion. Therefore, the majority of HMOs are neither absorbed nor metabolized in the proximal gut and reach the distal gut undigested to exert prebiotic effects on certain bacterial populations, to reinforce the intestinal barrier and to protect against enteropathogen infections (Licht et al., 2012; Valcheva and Dieleman, 2016; Barko et al., 2018). HMOs are present in human milk in a relatively high proportion (5–20 g/L). Their abundance changes over the course of lactation and their profile is highly variable among women because their structural diversity depends, inter alia, on genetic and geographical factors (Kulinich and Liu, 2016; McGuire et al., 2017). In contrast, the concentration of oligosaccharides in bovine milk, which is the basis of most infant formula, is 100- to 1000-fold lower than in human milk (Bode, 2015).

The production of single synthetic HMOs is increasingly becoming available for commercial purposes as their addition to infant formulas seems to be safe and beneficial for human infants and even for certain medical disorders (Donovan and Comstock, 2016; Xiao et al., 2017). The most abundant HMO is 2′-fucosyllactose (2′-FL), representing ~30% of the total oligosaccharides in human milk (He et al., 2016a). *In vitro* and *in vivo* studies with 2′-FL have evidenced its immunomodulatory effects, which include promoting Th2 and anti-inflammatory activities (He et al., 2016b), the inhibition of the colonization of pathogens (Ruiz-Palacios et al., 2003; Weichert et al., 2016; Yu et al., 2016) and an enhancement of cognitive abilities in rodents (Vázquez et al., 2015). Moreover, with the aim of mimicking the composition of human milk, a few clinical studies have tested the potential beneficial effects of feeding infants with a formula containing 2′-FL. The addition of 2′-FL was safe, well tolerated, the growth was comparable to breastfed infants (Marriage et al., 2015; Puccio et al., 2017) and it modified innate and adaptive immune profiles to be more like those of babies fed with human milk (Goehring et al., 2016). However, although 2′-FL has not proven a prebiotic function yet, it seems to be a good candidate (Gibson et al., 2017). Oligosaccharides with a proven prebiotic function are included in infant formulas. The addition of a mixture of short-chain galacto-oligosaccharides (scGOS) and long-chain fructo-oligosaccharides (lcFOS) at a 9:1 ratio in infant formulas has demonstrated multiple health-promoting effects: a reduction in the incidence of infections (Arslanoglu et al., 2007; Bruzzese et al., 2009), the modulation of the immune response (Bakker-Zierikzee et al., 2006; Vos et al., 2006, 2007; Scholtens et al., 2008; van Hoffen et al., 2009; Kostadinova et al., 2017) and changes in the microbiota composition (Scholtens et al., 2008; Huet et al., 2016).

Rotavirus (RV) is the main causative agent of acute diarrheal disease in infants, which can lead to dehydration or to more severe complications and even to death if left untreated (Hostetler, 2004). As the human RV pathogenesis is still unclear and RV vaccines are not globally implemented, the modulation by nutritional interventions with bioactive components is of interest (Gonzalez-Ochoa et al., 2017). Recent studies have suggested the possible role of both HMOs and GOS/FOS in the protection against RV-associated diarrhea (Comstock et al., 2017; Laucirica et al., 2017; Rigo-Adrover et al., 2017a,b), but several aspects regarding the mechanism of action involved in such protection are still a matter of study.

Therefore, the present study aimed to evaluate the activity of 2′-FL, scGOS/lcFOS 9:1 and their combination in modulating RV-induced diarrhea in a neonatal rat model. Moreover, we also aimed to further investigate the mechanisms of action involved, by assessing their effects on the intestinal barrier function and the gut and systemic immune responses.

## Materials and methods

### Animals

Fifteen G15 pregnant Lewis rats (LEW/OrlRj) were obtained from Janvier Labs (Le Genest-saint-Isle, France), individually housed in cages (2184L Eurostandard Type II L, Tecniplast, West Chester, PA, USA) with large fibrous particles bedding and tissue papers as enrichment, monitored daily and allowed to deliver naturally. The day of birth was registered as day 1 of life. On day 2, litters were randomly assigned to the experimental groups and culled to 8 pups per lactating dam, with free access to maternal milk and rat diet. Dams were given a commercial diet corresponding to the American Institute of Nutrition 93G formulation (Reeves et al., 1993; Teklad Global Diet 2014, Envigo, Indianapolis, IN, USA) and water *ad libitum*. Animal handling was performed during the first hours of the light phase on a scheduled basis, to avoid the influence of biological rhythms. After separating all the mothers and keeping the pups in the home-cage, handling and oral administration was performed. Afterwards, the dam was reunited with the whole litter. Animals were housed under controlled conditions of temperature and humidity in a 12 h light−12 h dark cycle, in a special isolated room designed and authorized to work under biosecurity level 2 conditions, in the Faculty of Pharmacy and Food Science animal facility (University of Barcelona, Spain).

All experimental procedures were conducted in accordance with the institutional guidelines for the care and use of laboratory animals and were approved by the Ethical Committee for Animal Experimentation of the University of Barcelona and the Catalonia Government (CEEA-UB Ref. 74/05 and DAAM 3046, respectively), in full compliance with national legislation following the EU-Directive 2010/63/EU for the protection of animals used for scientific purposes.

In order to calculate the sample size, the number of pups was used as the statistical unit. The number of animals in each group was established by the Appraising Project Office's program from the Universidad Miguel Hernández de Elche (Spain), which allowed the detection of statistically significant differences among groups assuming that there was no dropout rate and a type I error of 0.05 (two-sided). The variables used in the calculation included the clinical outcomes, such as the severity score. Moreover, independently of the number of animals obtained before, at least three litters were required for each group, as previous studies have demonstrated a remarkable variability between litters (Rigo-Adrover et al., 2017a). According to the estimation performed, 3 litters of 8 animals per group were sufficient. The final number of animals was not affected by the dropouts or outliers, which did not occur in the present study.

### Experimental design

Upon natural delivery, newborn rats were distributed into five groups of 24 animals each (3 litters of 8 animals): the reference (REF) group, rotavirus-infected (RV) group, and 3 rotavirus-infected groups supplemented with: (a) a mixture of scGOS and lcFOS in a 9:1 ratio (RV+GOS/FOS group); (b) 2′-FL (RV+2′-FL group); and (c) both scGOS/lcFOS and 2′-FL (RV+GOS/FOS+2′-FL group). All supplementations were provided by Nutricia Research (Utrecht, The Netherlands).

Suckling rats were orally administered once daily, as previously described (Rigo-Adrover et al., 2017b), with the same normalized volume/body weight of all products (4.5 μL/g/day), from the second to the sixteenth day of life, corresponding to the strict lactation period. The RV+GOS/FOS group was supplemented with 0.8 g of scGOS/lcFOS/100 g of body weight. The RV+2′-FL group was supplemented with 0.2 g of 2′-FL/100 g of body weight. 2′-FL was produced by microbial fermentation, with >90% purity. The RV+GOS/FOS+2′-FL group received both products at the same doses as when given separately and maintaining the volume of administration (4.5 μL/g/day). Finally, the REF and RV groups were administered with a matched volume of water.

The RV (simian SA-11) was obtained by the Virus Enteric Group of the University of Barcelona leaded by Dr. A. Bosch and Dr. R. Pintó under the compliance of the current principles of GLP (Royal Decree 1369/2000, July 19); the viruses were propagated in fetal African green monkey kidney (MA-104) cells and titered as plaque-forming units (PFUs), as previously described (Pérez-Cano et al., 2007). The virus was inoculated at day 5 of life (4 × 10^8^ Tissue Culture Infectious Dose 50 [TCID50]/rat) in all the experimental groups with the exception of the REF group, which received the same volume of phosphate-buffered solution (PBS) under the same conditions.

Body weight was recorded daily throughout the study to assess weight gain. Half (*n* = 12) of each group of animals were sacrificed at day 8, to analyze variables associated with the peak of diarrhea, and the other half (*n* = 12 per group) at day 16, to analyze the effects of the supplementations once the diarrhea was resolved. Moreover, the naso–anal and tail lengths were measured to determine the body/tail ratio, the body mass index (BMI), calculated as body *weight/length*^2^ (g/cm^2^) and the Lee Index, calculated as (*weight*^0.33^*/length) x 1000* (g^0.33^/cm).

### Fecal samples collection and clinical indices

Fecal sampling was performed once daily throughout the study (from day 4 to day 16 of life) by gently pressing and massaging the abdomen. Fecal samples were stored at −20°C for the analysis of RV shedding. Severity of diarrhea was expressed by fecal weight and by scoring fecal samples from 1 to 4 (diarrhea index [DI]) based on color, texture and amount, as follows: normal feces (1); soft yellow-green feces (2); totally loose yellow-green feces (3); high amount of watery feces (4). Scores ≥2 indicate diarrheic feces, whereas scores < 2 indicate absence of diarrhea (Pérez-Cano et al., 2007). The severity-area under the curve (S-AUC) during days 5–11, coinciding with the period in which animals displayed diarrhea, was calculated as a global value of severity. The maximum severity (MS) was defined as the highest score during the diarrhea period.

Incidence of diarrhea was expressed by the percentage of diarrheic animals (%DA), which was based on the percentage of animals displaying scores of DI ≥2 in each group. The incidence-area under the curve (I-AUC) during days 5–11, coinciding with the period in which animals displayed diarrhea, was calculated as a global value of incidence. The maximum incidence (MI) was defined as the highest %DA during the diarrhea period. The diarrhea period (DP) was calculated by counting the number of days in which the animals displayed DI ≥2.

The DI, S-AUC, MS, %DA, I-AUC MI and DP were normalized (nDI, nS-AUC, nMS, n%DA, nI-AUC, nMI and nDP) in RV+GOS/FOS and RV+GOS/FOS+2′-FL groups, because of intrinsic fecal aspects of GOS/FOS supplementation, as previously described (Rigo-Adrover et al., 2017b). The data from REF, RV and RV+2′-FL were not normalized, because no basal effect was observed. The normalization of data was performed by calculating the mean DI of the timepoints when there was no active diarrhea in the RV group (e.g., before the infection, days 4–5, and once the diarrhea is solved, days 12–17). Finally, the difference between this mean and the baseline score (DI = 1) was subtracted to all values of DI. All in all, to ascertain the effects of GOS/FOS and GOS/FOS+2′-FL on RV infection, only the normalized data must be read.

### Sample collection and processing

At days 8 and 16, half of each litter were intramuscularly anesthetized with ketamine (90 mg/kg) (Merial Laboratories S.A., Barcelona, Spain) and xylazine (10 mg/kg) (Bayer A.G., Leverkusen, Germany), exsanguinated and their thymus, spleen, liver and intestines were obtained to record the weight. Two 1 cm sections of the central part of the small intestine were collected for histomorphometric and gene expression analysis. The remaining parts of the intestine were opened lengthwise, cut into 5 mm pieces and incubated with 2 mL of PBS in a shaker (10 min, 37°C) to obtain the gut wash (GW). After centrifugation, supernatants were stored at −20° and −80°C until alpha-1 antitrypsin (A1AT) and cytokine analysis, respectively. Plasma was obtained after blood centrifugation and kept at −20°C for immunoglobulin analysis.

### Small intestine histology

Fixed intestinal tissues corresponding to the peak of RV infection (day 8) were dehydrated, paraffin-embedded and cut into 5 μm cross sections using a microtome (RM2135, Leica, Wetzlar, Germany). Subsequently, the sections were mounted on glass slides and kept until staining with hematoxylin-eosin. Finally, slides were mounted with coverslips using ProLong™ Gold Antifade Mountant (Life Technologies, Carlsbad, CA, USA) and dried overnight. The observation of the intestinal architecture was performed using a bright-field microscope (Olympus BX41, Olympus Corporation, Shinjuku, Tokyo, Japan) at 100x. The sample size was 6 animals, representative of the three litters in each experimental group (2 animals/litter). The intestinal perimeter was calculated by measuring the outer layer of the intestine. Seven villi were selected randomly in each animal and the villi height and area were measured. The villi width was measured at the junction of villi and crypts. The crypts depth and the villi height/crypts depth ratio were also analyzed. The statistical analysis was performed with each animal's mean value of the variables described above. All morphometric measures were performed with ImageJ (Image Processing and Analysis in Java, National Institute of Mental Health, Bethesda, MD, USA).

### Fecal SA11 shedding by elisa

Fecal samples from day 6 (1 day post-inoculation [DPI]) were diluted in PBS (10 mg/mL) and homogenized using Pellet Pestles Cordless Motor (Sigma-Aldrich, Madrid, Spain). Homogenates were centrifuged (170 *g*, 5 min, 4°C) and supernatants were frozen at −20°C until analysis. SA11 virus particles were quantified by ELISA, as previously described (Rigo-Adrover et al., 2017a). Titrated dilutions of inactivated SA11 particles, ranging from 10^6^ to 10^4^/mL, were used as standard curve.

### *In vitro* blocking assay

With the objective of testing the ability of the assayed oligosaccharides to bind the RV, an in-house *in vitro* blocking assay was performed (Rigo-Adrover et al., 2017b). SA11 stock was diluted with 1% PBS-Tween to reach a concentration of 5 × 10^4^ viral particles/mL, which is the highest concentration previously found in the feces of infected animals in this model. Starting from the *in vivo*-administered concentration, different dilutions (from 1/2 to 1/16) of GOS/FOS or 2′-FL were preincubated with the virus at 1/1 ratio for 30 min. After that, free, noncoated viral particles were quantified by ELISA, as described in section Fecal SA11 shedding by ELISA. In order to demonstrate that the products were directly binding to virions, a negative control was used. The products were added to anti-RV Ab coated wells (without the presence of the virus) and after their incubation during 30 min, the supernatants were removed. At that point, the standard virus was added. Their levels were not affected with respect to those in which the product was not added previously, meaning that the oligosaccharides are not binding to the Ab, at least in a critical position.

### Intestinal permeability assay

The quantification of A1AT in the gut wash, as a marker of intestinal permeability, was performed with the rat SERPINA1/Alpha 1 Antitrypsin ELISA kit (LifeSpan Biosciences Inc., Seattle, WA, USA) following the manufacturer's instructions. The standard concentrations ranged from 100 to 1.563 ng/mL. Assay sensitivity was 1.56 ng/mL.

### Immunoglobulins in plasma

Plasma concentrations of IgM, IgG1, IgG2a, IgG2b, IgG2c, and IgA at the end of the nutritional intervention (day 16) were quantified using ProcartaPlex™ Multiplex immunoassay (eBioscience, San Diego, CA, USA). In brief, specific color-coded capture beads were bound to the analyte of interest. After adding different detection antibodies conjugated to phycoerythrin (PE), the specific concentration of each analyte was obtained by MAGPIX® analyzer (Luminex Corporation, Austin, TX, USA) at the Scientific and Technological Centers of the University of Barcelona (CCiT-UB). Assay sensitivity was as follows: 0.02 ng/mL for IgM; 0.78 ng/mL for IgG1; 0.02 ng/mL for IgG2a; 0.11 ng/mL for IgG2b; 0.19 pg/mL for IgG2c and 0.48 pg/mL for IgA. Anti-RV total and IgM immunoglobulins in plasma were quantified by ELISA as in previous studies (Rigo-Adrover et al., 2017b).

### Intestinal cytokines in gut wash

Gut wash concentrations of interleukin (IL)-1α, IL-4, IL-6, IL-10, IL-12p70, interferon (IFN)-γ and tumor necrosis factor (TNF)-α at both day 8 and 16 of the study were quantified using ProcartaPlex™ Multiplex immunoassay (eBioscience). In brief, specific color-coded capture beads were bound to the analyte of interest. Then, different detection antibodies conjugated to biotin were added. After incubating with streptavidin-PE, the specific concentration of each analyte was obtained using a MAGPIX® analyzer (Luminex Corporation) at the CCiT-UB. Assay sensitivity was as follows: 3.5 pg/mL for IL-1α; 0.1 pg/mL for IL-4; 1.9 pg/mL for IL-6; 1.6 pg/mL for IL-10; 5.9 pg/mL for IL-12p70; 0.6 pg/mL for IFN-γ; and 0.4 pg/mL for TNF-α.

### Intestinal gene expression by real-time PCR

The central section of the small intestine was homogenized for 30 s in lysing matrix tubes (MP Biomedicals, Illkirch, France) using a FastPrep-24 instrument (MP Biomedicals), as previously described (Camps-Bossacoma et al., 2017). RNA was isolated with the RNeasy® Mini Kit (Qiagen, Madrid, Spain) following the manufacturer's instructions. RNA purity and concentration were determined with a NanoPhotometer (BioNova Scientific S.L., Fremont, CA, USA). Later, cDNA was obtained in a thermal cycler PTC-100 Programmable Thermal Controller using TaqMan® Reverse Transcription Reagents (Applied Biosystems, AB, Weiterstadt, Germany).

The specific PCR TaqMan® primers (AB) used to assess gene expression with real-time PCR (ABI Prism 7900 HT, AB) were *pIgR* (Rn00562362_m1, I), *Iga* (4331348, made to order), *Ifng* (Rn00594078_m1, I), *Tnf* (Rn99999017_m1, I), *Tgfb1* (Rn00572010_m1, I), *Il10* (Rn00563409_m1, I), *Il17a* (Rn01757168_m1, I), *Il22* (Rn01760432_m1, I), *Muc2* (Rn01498206_m1, I), O*cln* (Rn00580064_m1, I), *Cldn2* (Rn02063575_s1, I), *Fcgrt* (Rn00583712_m1, I, encoding for FcRn), and *Prdm1* (Rn03416161_m1, I, encoding for Blimp-1). The relative gene expression was normalized with the housekeeping gene *Gusb* (Rn00566655_m1, I) using the 2 ^−ΔΔCt^ method (Livak and Schmittgen, 2001). Results are expressed as percentage of values of each experimental group normalized to the mean value obtained for the REF group, which was set at 100%.

### Short-chain fatty acids in the cecal content

Short-chain fatty acid (SCFA) quantification in cecal content from 16-day-old rats was performed by headspace-gas chromatography-mass spectrometry (HS-GC-MS) at the GC-MS unit of the CCiT-UB. Samples were acidified in 0.1 M formic acid and homogenized using Pellet Pestles Cordless Motor (Sigma-Aldrich) to reach a concentration of 200 mg/mL. Then, 100 μL of the homogenized cecal content was added to 20 μL of internal standard (2-ethylbutyric 85 μM) in a 10 mL headspace vial (Agilent, Santa Clara, CA, USA). After incubating the sample for 20 min at 60°C, 1 mL of the headspace gap was injected by a Triplus HS autosampler (Thermo Fisher Scientific, Barcelona, Spain) into a TRACE GC Ultra system (Thermo Fisher Scientific) configured with an inlet split flow of 10 mL/min. The separations were performed using an HP-FFAP column (25 m × 0.2 mm I.D × 0.3 μm film thickness) (Agilent) with an injector temperature of 250°C and an oven temperature program using two rates: from 80°C (3 min) up to 125°C at 50°C/min, and from 125°C up to 225°C at 6°C/min. The carrier gas was helium with a flow of 1 mL/min and the GC-MS transfer line temperature was 250°C. The MS was a DSQII (Thermo Fisher Scientific), which operated in electron ionization and selected ion monitoring scanning mode (masses 43, 45, 57, 60, 71, 73, 74, 87, 88, and 116). A 10 mM volatile free acid mix serial dilution (Supelco, Bellefonte, PA, USA) containing formic, acetic, propionic, isobutyric, butyric, isovaleric, valeric, isocaproic, caproic and heptanoic acids was used as standard. All analyses were performed in triplicate. The identification of each compound was carried out using the spectra obtained with standard compounds and from the NIST mass spectral library. The internal standard method of quantification was used for all the targeted acids with the exception of acetic acid, the quantification of which was performed by the external standard method (reduced variability was observed). No relevant matrix effect was detected. The lower limits of detection (in μmol/g of feces) were as follows: 0.404 for acetic acid, 0.068 for propionic acid, 0.003 for isobutyric acid, 0.020 for butyric acid, 0.001 for isovaleric acid, 0.001 for valeric acid, 0.025 for isocaproic acid, 0.012 for caproic acid and 0.046 for heptanoic acid.

### Statistical analysis

The Statistical Package for the Social Sciences (SPSS v22.0) (IBM, Chicago, IL, USA) was used for statistical analysis. Data was tested for homogeneity of variance and normality distribution by the Levene's and Shapiro–Wilk tests, respectively. When data was homogeneous and had a normal behavior, conventional one-way ANOVA test followed by the *post hoc* Bonferroni was performed. Otherwise, the nonparametric Kruskal–Wallis test followed by the *post hoc* Mann–Whitney U (MWU) test were performed. Finally, the chi-square test was used to compare frequencies of diarrhea incidence. Significant differences were established when *p* < 0.05.

## Results

### Growth and morphometry

The RV infection did not produce any significant change in growth either at the peak of diarrhea (day 8) or at the end of the study (day 16), as shown by the results in body weight, body/tail ratio, BMI and Lee Index (Table 1). The group supplemented with GOS/FOS had a slightly higher body weight at the end of the study (day 16, *p* < 0.05), and although none of these growth changes modified the BMI, some differences were seen in the body/tail length ratio and the Lee Index. All supplementations increased the body/tail length ratio compared to REF or RV at some time point. Moreover, the Lee Index was decreased exclusively at the peak of diarrhea compared to REF.

**Table 1:** Growth-associated variables.

		**REF**	**RV**	**RV+GOS/FOS**	**RV+2′-FL**	**RV+GOS/FOS+2′-FL**
**Body weight (g)**						
	d8	13.52 ± 0.25	13.60 ± 0.19	14.08 ± 0.17	13.51 ± 0.19	13.67 ± 0.19
	d16	31.40 ± 0.89	31.91 ± 0.65	34.16 ± 0.55^*^^#^	32.79 ± 0.50	33.53 ± 0.40
**Body/tail length ratio**						
	d8	2.14 ± 0.07	2.15 ± 0.03	2.26 ± 0.03^*^^#^	2.32 ± 0.03^*^^#^	2.22 ± 0.03
	d16	1.77 ± 0.02	1.77 ± 0.02	1.92 ± 0.04^*^^#^	1.86 ± 0.05	1.84 ± 0.03^*^
**BMI (g/cm**^2^**)**						
	d8	0.30 ± 0.01	0.29 ± 0.00	0.29 ± 0.00	0.28 ± 0.01	0.28 ± 0.00
	d16	0.35 ± 0.01	0.36 ± 0.00	0.37 ± 0.01	0.37 ± 0.01	0.36 ± 0.00
**Lee index (g**^0.33^**/cm, x1000)**						
	d8	354.76 ± 3.42	348.80 ± 1.73	345.21 ± 1.34^*^	343.49 ± 2.46^*^^#^	344.52 ± 1.43^*^
	d16	334.44 ± 2.51	335.88 ± 2.41	337.12 ± 3.36	337.93 ± 3.48	332.44 ± 1.26
**SI/body (w/w, %)**						
	d8	3.10 ± 0.06	3.35 ± 0.04^*^	4.56 ± 0.11^*^^#^	3.84 ± 0.10^*^^#^	4.26 ± 0.10^*^^#^
	d16	2.88 ± 0.06	2.88 ± 0.06	3.99 ± 0.09^*^^#^	3.00 ± 0.07	3.58 ± 0.21^*^^#^
**LI/body (w/w, %)**						
	d8	0.60 ± 0.01	0.60 ± 0.03	0.68 ± 0.02	0.69 ± 0.03	0.75 ± 0.02^*^^#^
	d16	1.07 ± 0.03	0.98 ± 0.02^*^	1.25 ± 0.06^*^^#^	0.95 ± 0.07^*^	1.24 ± 0.05^*^^#^

*Results are expressed as mean ± S.E.M. (n = 12)*.

* *p < 0.05 compared to REF (by MWU test)*.

# *p < 0.05 compared to RV (by MWU test)*.

*BMI, Body mass index; SI, small intestine ; LI, large intestine; w/w, weight/weight; day 8, d8; day 16, d16*.

With regard to organ weight, no changes were observed in the relative weight of the thymus and liver (data not shown), whereas a slight increase in the spleen was found for RV+GOS/FOS and RV+2′-FL at day 16 (0.53 ± 0.03 and 0.53 ± 0.02, respectively) compared with that of RV (0.47 ± 0.02, *p* < 0.05). RV and oligosaccharide supplementations changed the relative small and large intestine weight, which in some cases had a larger percentage of the total body weight (Table 1). At day 8, the relative small intestine weight increased in the RV group (*p* < 0.05) and it was even higher in the oligosaccharide groups (*p* < 0.05), which also showed higher values on day 16 (with the exception of the RV+2′-FL group). These changes for RV+GOS/FOS and RV+GOS/FOS+2′-FL were also observed in the large intestine, whereas the RV+2′-FL group showed a lower large intestine weight.

### Histomorphometric analysis and maturation markers in the small intestine

**Figure 1:**
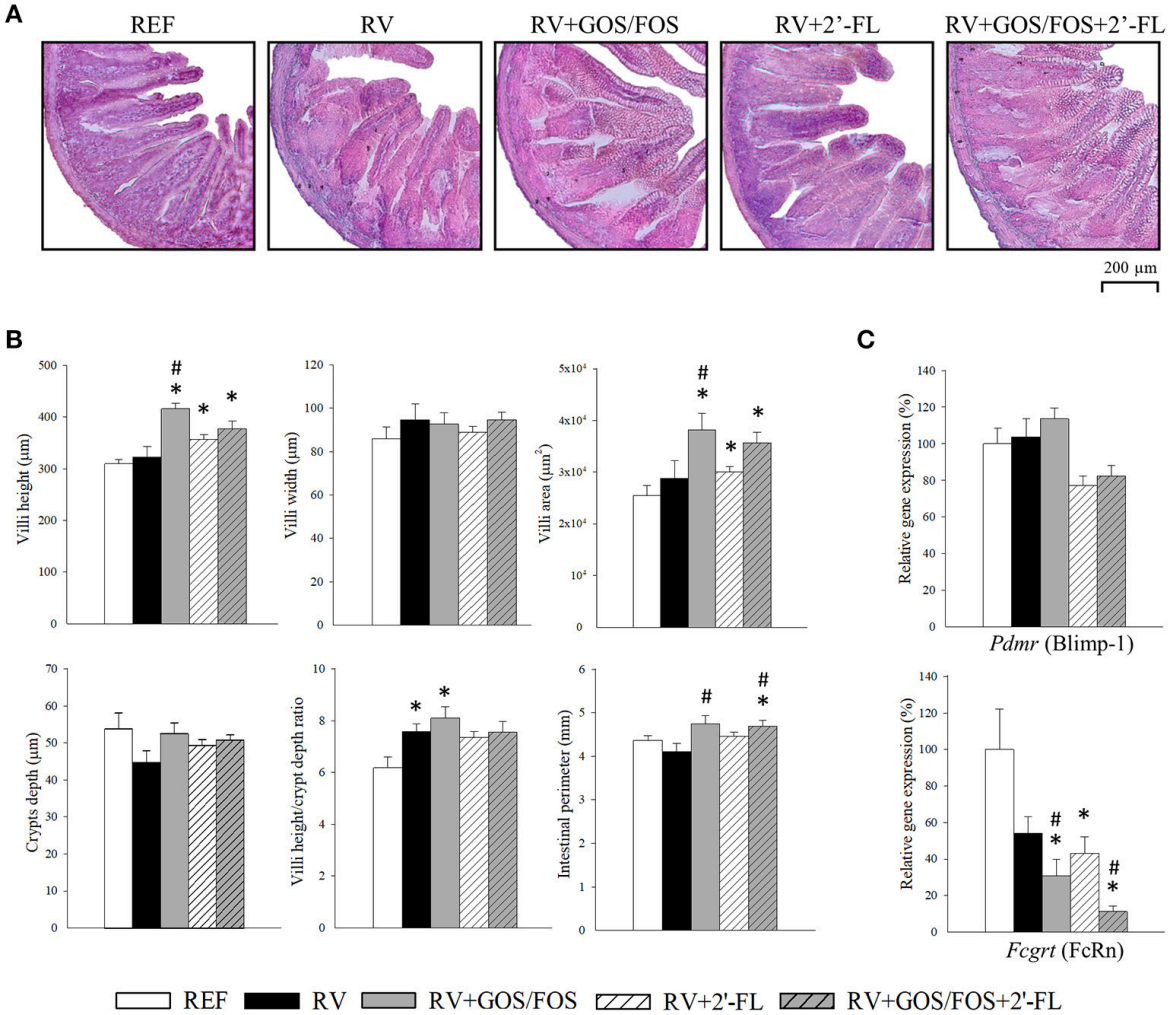
Effect of GOS/FOS, 2′-FL and their combination on histomorphometric variables and the intestinal gene expression of maturation markers at the peak of diarrhea (day 8). **(A)** Representative images of histological sections of the jejunum stained with hematoxylin and eosin, 100X. **(B)** Villi height, villi width, villi area, crypts depth, villi height/crypts depth ratio and perimeter of the jejunum of suckling rats. **(C)** Relative gene expression of the maturation markers *Pdmr* (encoding for Blimp-1) and *Fcgrt* (encoding for FcRn) by real-time PCR. The relative gene expression was calculated with respect to REF, which corresponded to 100% of transcription. Results are expressed as mean ± S.E.M. (*n* = 4–8). *p < 0.05 compared to REF group; ^#^*p* < 0.05 compared to RV group (by MWU test).

The analysis of the jejunum intestinal architecture was performed at day 8 (Figure 1), corresponding to the peak of diarrhea and allowing the observation of changes induced by both the RV and the nutritional supplementations. In our study, we did not detect major histological differences due to the RV infection compared to the REF group (Figure 1A). Specifically, the RV group did not show visual changes of vacuolization, swelling of villi tips or constriction of the bases, as reported for other RV strains (Boshuizen et al., 2003). Moreover, SA11 did not induce villus atrophy or intestinal swelling, as the villus height, area and the intestinal perimeter were similar to those of the REF group (Figure 1B). Nevertheless, the RV group showed an increase in the villi height/crypts depth ratio (*p* < 0.05), although no differences were detected in the absolute measure of villus height or crypt depth.

The supplementations were able to promote positive changes in some of the morphometric variables analyzed. All rats supplemented with any of the oligosaccharides displayed higher villi height (Figure 1B) and area compared to REF, especially those groups supplemented with GOS/FOS, whose villi were more than 30% higher than those of the REF and RV groups (*p* < 0.05). No changes were observed in the measure of villi width in any of the groups. Furthermore, the perimeters of the intestines of GOS/FOS-supplemented animals were found to be greater than those in the RV group (*p* < 0.05), confirming an intestinal trophic effect for these oligosaccharides (Figure 1B).

The expression of the genes encoding for FcRn and Blimp-1 (Figure 1C), enables the intestinal maturation process in suckling rats to be established (Arévalo Sureda et al., 2017). Although the inoculation with RV did not affect Blimp-1, it showed a trend to reduce the expression of FcRn in the RV-infected groups, probably indicating an accelerated intestinal maturation due to the challenge. However, in the RV+GOS/FOS and RV+GOS/FOS+2′-FL groups, its expression was reduced to a greater extent compared to RV (*p* < 0.05), suggesting that probably GOS/FOS is involved in intestinal maturation. No differences were found in the expression of Blimp-1 after any of the supplementations (Figure 1C).

### Clinical variables for the assessment of severity, incidence and duration of diarrhea

**Figure 2:**
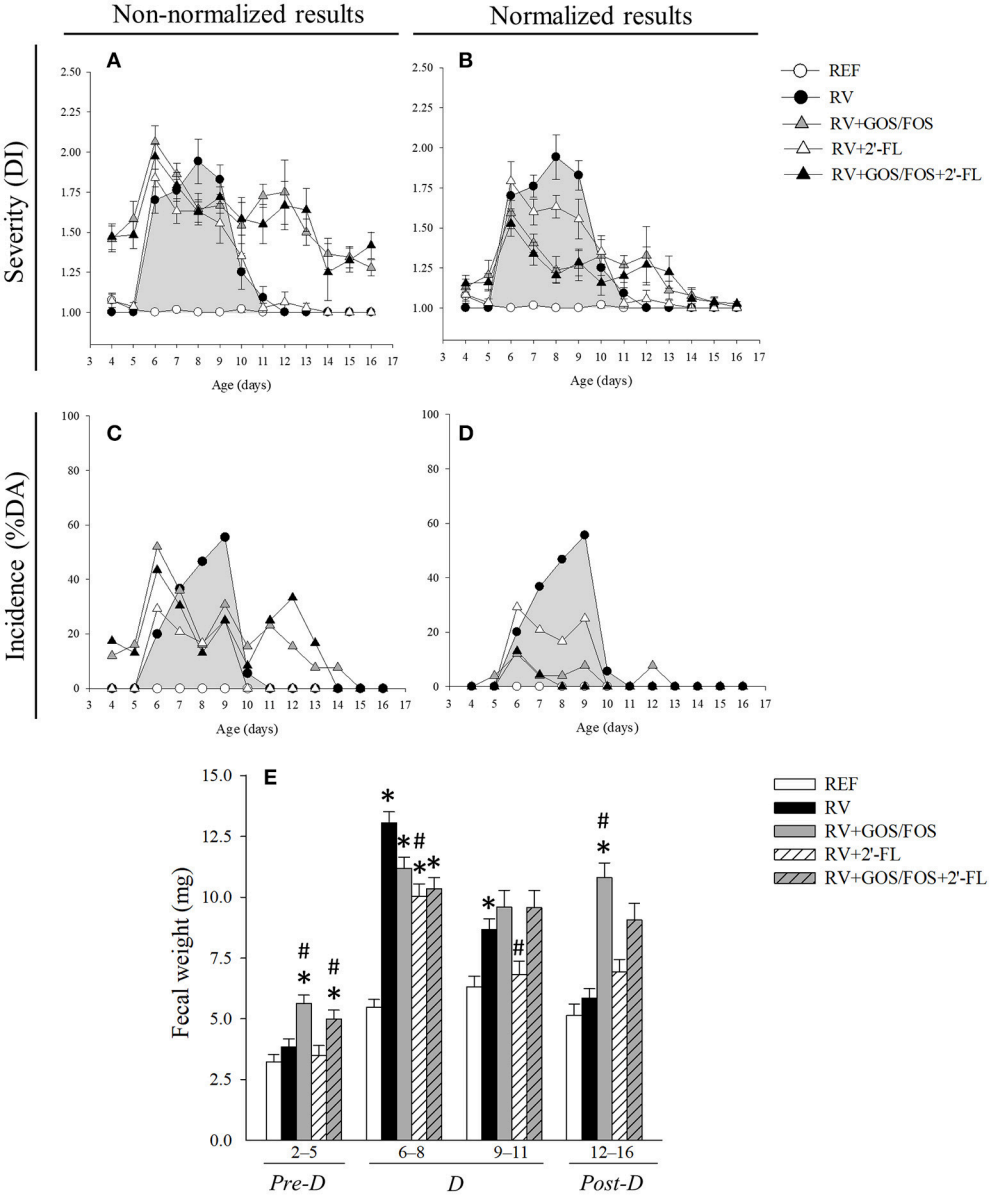
Clinical indices of diarrhea. **(A,B)** The severity of diarrhea is represented with the Diarrhea Index (DI), which is based on scoring fecal samples from 1 to 4 depending on the color, texture and abundance. Scores of DI ≥2 indicate presence of diarrhea, whereas scores < 2 indicate absence of diarrhea. **(C,D)** The incidence of diarrhea is represented with the percentage of diarrheic animals (%DA), which is based on the percentage of animals displaying DI scores ≥2 in each group. **(A,C)** Data is shown non-normalized or **(B,D)** normalized for the RV+GOS/FOS and RV+GOS/FOS+2′-FL groups taking into account basal values. **(E)** The fecal weight, as an objective indicator of the severity of diarrhea, was calculated as the mean value in the pre-diarrhea, diarrhea and post-diarrhea periods. Results are expressed as mean ± S.E.M. for severity (*n* = 3–24, depending on the number of fecal samples obtained each day) and unique values for incidence (derived from all animals each day). Statistical differences not shown in **(A–D)**. *p < 0.05 compared to REF group; ^#^*p* < 0.05 compared to RV group (by MWU test).

Rats inoculated with RV displayed diarrheic feces from days 6 to 11 of life, the highest score being at day 8 (Figures 2A,B). Most of the animals in the RV group (21/24, 87.5%) displayed diarrhea (DI ≥2) at some timepoint, although when calculating the mean DI day by day its value was lower than 2. As shown in a previous study using the SA11 RV infection model, GOS/FOS induced changes in fecal consistency, thereby increasing the number of feces considered as diarrheic before, during and after the diarrheic process (Rigo-Adrover et al., 2017b). For this reason, in order to ascertain the effects of GOS/FOS and GOS/FOS+2′-FL on RV infection, only the normalized data must be read (Figures 2B,D). The severity of diarrhea of animals which received these two supplementations showed a significant reduction (*p* < 0.05) at days 7, 8 and 9 (Figure 2B). The supplementation with 2′-FL did not induce this basal fecal consistency effect and displayed a trend to reduce the severity of diarrhea (Figure 2A).

The incidence of diarrhea, calculated as the percentage of diarrheic animals (%DA), evidenced clearer effects (Figures 2C,D). The RV group displayed the highest incidence of diarrhea on day 9, when 55.5% of the animals were scored DI ≥2. However, all three oligosaccharide supplementations induced amelioration throughout the entire diarrhea period (Figure 2C), attaining statistical significance at day 8, when less than 20% of the animals had symptoms of diarrhea (*p* < 0.05). After the normalization of data in the groups supplemented with GOS/FOS (Figure 2D), the incidence of diarrhea in such groups was reduced by up to 10% DA in the course of the disease.

Other indicators of severity, incidence and duration of diarrhea were also calculated (Table 2). Regarding the maximum severity (MS) of diarrhea, the supplementation with 2′-FL and 15% compared to that of the RV group (*p* < 0.05). The normalized maximum severity (nMS), displayed a milder score for the GOS/FOS groups, showing a 28–30% reduction (*p* < 0.05). The calculation of the severity-area under the curve (S-AUC) displays the average severity for each group. A clear tendency to reduce the S-AUC with respect to the RV group was seen for the supplementation with 2′-FL, with a 22% decrease. The normalized area under the curve (nS-AUC) was reduced in the animals supplemented with GOS/FOS and GOS/FOS+2′-FL (45 and 58%, respectively, *p* < 0.05). Similar results were seen when calculating incidence indicators. The maximum incidence (MI) and incidence-area under the curve (I-AUC) were reduced in all groups compared to the RV group, displaying up to 45% of amelioration upon 2′-FL supplementation and 80 and 90% for normalized severity indicators in GOS/FOS and GOS/FOS+2′-FL, respectively (*p* < 0.05). Finally, the amelioration was also found in terms of reducing the diarrhea period (DP), which was reduced more than 50% due to the supplementations (*p* < 0.05).

**Table 2:** Analysis of other variables associated with severity, incidence and duration of diarrhea.

	**RV**	**RV+GOS/FOS**	**RV+2′-FL**	**RV+GOS/FOS+2′-FL**
**SEVERITY**				
MS	2.23 ± 0.07	2.09 ± 0.10	1.88 ± 0.09^#^	2.01 ± 0.07^#^
nMS	–	1.61 ± 0.10^#^	–	1.55 ± 0.07^#^
S-AUC	2.85 ± 0.17	3.56 ± 0.31	2.22 ± 0.35	2.94 ± 0.33
nS-AUC	–	1.57 ± 0.19^#^	–	1.18 ± 0.21^#^
**INCIDENCE**				
MI	55.56	52.00	29.17^a^	43.48
nMI	–	12.00^#^	–	13.04^#^
I-AUC	164.44	169.69	91.67	139.31
nI-AUC	–	29.69	–	17.39
**DURATION**				
DP	1.40 ± 0.15	1.84 ± 0.19	0.79 ± 0.16^#^	1.74 ± 0.20
nDP	–	0.32 ± 0.13^#^	–	0.17 ± 0.08^#^

*Results are expressed as mean ± S.E.M. for severity and duration variables (n = 12–24 depending on the number of fecal samples obtained each day) and unique values for incidence variables (derived from all animals each day)*.

# *p < 0.05 compared to RV (by MWU test and Chi Square test); MS, maximum severity; nMS, normalized MS; S-AUC, severity-area under the curve; nS-AUC, normalized S-AUC; MI, maximum incidence; nMI, normalized MI; I-AUC, incidence-area under the curve; nI-AUC, normalized I-AUC*.

a *p = 0.08*.

The fecal weight was also measured as an objective severity variable of the diarrheic process (Figure 2E). Before RV inoculation (pre-diarrhea period [pre-D], corresponding to days 2–5 of life), animals receiving GOS/FOS and GOS/FOS+2′-FL had a higher fecal weight compared to both the REF and RV groups, which is likely to be related to the previously direct changes reported in the fecal consistency (Rigo-Adrover et al., 2017b). After infecting with the virus (diarrhea period, [D], corresponding to days 6–11 of life), the RV group showed an increased fecal weight (*p* < 0.05, Figure 2E). The effects of the supplementations with oligosaccharides were seen at days 6–8, reducing this increase of fecal weight, although only statistical differences in the supplementation with 2′-FL were attained (*p* < 0.05). This lower fecal weight observed in the 2′-FL group, was maintained until the end of the diarrhea period (days 9–11). At the end of the diarrheic process (post-diarrhea period, [post-D], days 12–16 of life), fecal weights from RV and 2′-FL were similar to those from REF, while the supplementation with GOS/FOS and GOS/FOS+2′-FL continued to show higher values due to the non-pathogenic changes in the fecal consistency.

### Viral shedding and oligosaccharide blocking activity

**Figure 3:**
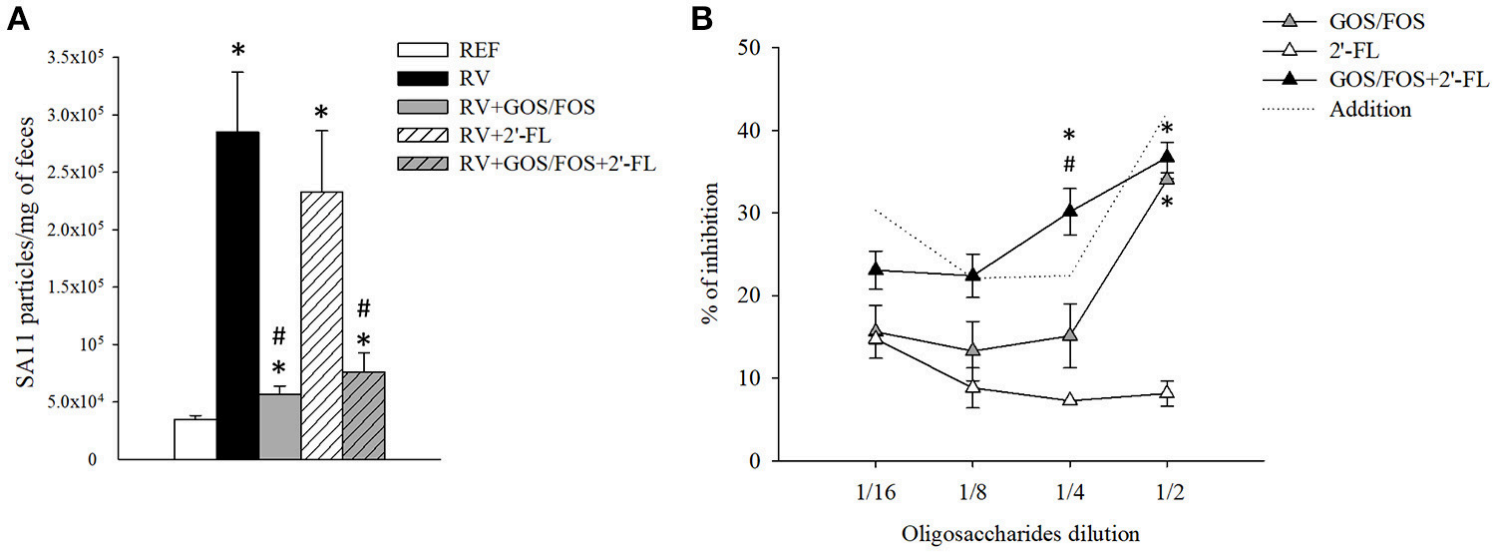
Viral shedding and *in vitro* blocking activity of the oligosaccharides used in this study. **(A)** The viral shedding in feces was assessed at the peak of viral elimination, corresponding to day 6. **(B)** The blocking activity of the oligosaccharides was tested by an in-house *in vitro* blocking assay using the ELISA technique. SA11 stock was diluted with PBS-Tween 1% to reach a concentration of 5 × 10^4^ viral particles/mL. Starting from the *in vivo*-administered concentration, different dilutions (from 1/2 to 1/16) of GOS/FOS, 2′-FL and GOS/FOS+2′-FL were preincubated with the virus at 1/1 ratio for 30 min. Free, non-coated viral particles were quantified by ELISA. The dotted line represents the addition of the results of the GOS/FOS and 2′-FL analyzed separately. Results are expressed as mean ± S.E.M. (*n* = 8 for viral shedding and *n* = 2 replicates from 2 experiments for the blocking assay). *p < 0.05 compared to REF group; ^#^*p* < 0.05 compared to RV group (by MWU test).

The viral shedding in feces (Figure 3A) was determined by ELISA at 1 DPI, which corresponded to the day of maximum elimination (Rigo-Adrover et al., 2017a). The RV group eliminated 2.9 × 10^5^ RV particles/mg of feces, similar to the group supplemented with 2′-FL, which eliminated 2.3 × 10^5^ RV particles. However, the RV particles detected in feces of the groups supplemented with GOS/FOS and GOS/FOS+2′-FL were drastically decreased (*p* < 0.05). In fact, this effect has been previously reported and could be linked to a GOS/FOS blocking activity, interfering with the quantification of viral particles by ELISA (Rigo-Adrover et al., 2017b). In this regard, to assess the binding activity of GOS/FOS and 2′-FL, an in-house *in vitro* blocking assay was performed (Figure 3B). As seen previously for GOS/FOS (Rigo-Adrover et al., 2017b), there was a dose-dependent inhibition of RV detection after preincubating with the same mixture of oligosaccharides, reaching an inhibition of approximately 30–40%. Moreover, the 2′-FL also showed the blocking activity to a lesser extent, with up to 10–20% of inhibition. Interestingly, the combination of both oligosaccharides elicited an additive blocking activity, as seen by an inhibition of RV detection similar to the sum of the inhibitions of the two oligosaccharides.

### Intestinal barrier function

**Figure 4:**
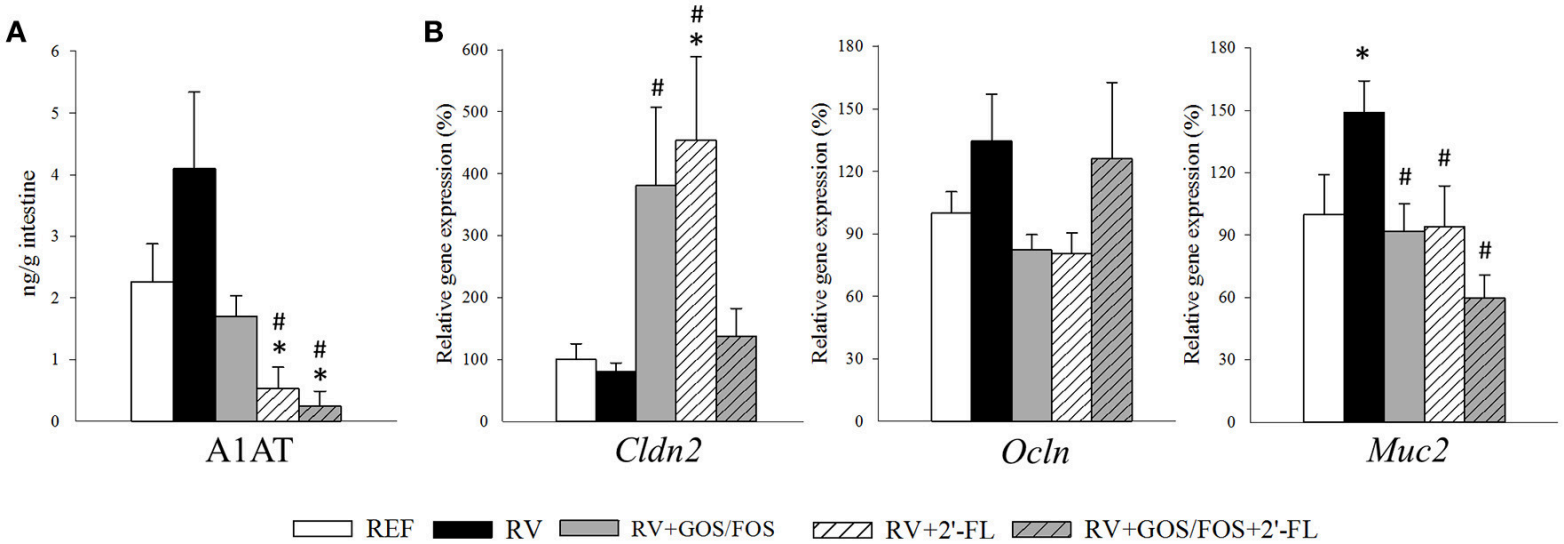
Assessment of oligosaccharide supplementation on the intestinal barrier function. The effects on the intestinal barrier function was studied at the peak of diarrhea (day 8). **(A)** The alpha-1 antitrypsin (A1AT) concentration in the gut wash was analyzed by ELISA as a measure of the intestinal barrier disruption. **(B)** Relative expression of tight junction molecules *Cldn2* and *Ocln* and the mucin Muc2 were quantified by real-time PCR. Relative gene expression was calculated with respect to REF, which corresponded to 100% of transcription. Results are expressed as mean ± S.E.M. (*n* = 4–8). *p < 0.05 compared to REF group; ^#^*p* < 0.05 compared to RV group (by MWU test).

The A1AT levels in the gut wash at day 8 of life (Figure 4A) were determined to assess the changes in the gut permeability. The plasma level of A1AT is significantly elevated during the inflammatory response and can be transported across the intestinal epithelial layer into the intestinal lumen due to the increased permeability of the intestinal epithelial barrier (Yang et al., 2015). The RV group displayed higher levels of A1AT in the gut wash, but they did not differ statistically from the REF group. However, the supplementation with 2′-FL and GOS/FOS+2′-FL reduced the concentration of A1AT in the gut wash compared to both the RV and REF (*p* < 0.05), indicating that not only they were able to prevent the gut permeability disruption induced by RV, but also promoted an enhanced gut barrier function.

The gene expression of molecules involved in the intestinal barrier function, such as the tight junctions (TJ) or mucin was assessed by real-time PCR at day 8 of life (Figure 4B). The expression of occludin (*Ocln*) and claudin 2 (*Cldn2*) were not altered due to RV infection, yet the supplementations with GOS/FOS and 2′-FL—but not their combination—raised the expression of *Cldn2* (*p* < 0.05). Regarding the goblet cell-specific mucin 2 (*Muc2*), which is involved in the active defense against RV infection (Boshuizen et al., 2005), in our study its expression was increased at day 8 in the RV group (*p* < 0.05). Such increase was not found in the groups infected with RV and supplemented with the oligosaccharides, whose levels were similar to those of the REF group.

### Antibody production

There is evidence supporting the potential of HMOs to directly affect immune activity in RV-infected neonates (Comstock et al., 2017). For this reason, we assessed the levels of antibodies in the plasma of the suckling rats at the end of the study (day 16, Table 3). The total and IgM anti-RV antibody levels were not modified due to SA11 infection, although a tendency to increase anti-RV IgM in the RV group and in those groups supplemented with GOS/FOS was observed.

**Table 3:** Analysis of the concentration of anti-RV antibodies and the concentration of immunoglobulins in plasma at the end of the study (d16).

		**REF**	**RV**	**RV+GOS/FOS**	**RV+2′-FL**	**RV+GOS/FOS+2′-FL**
**Anti-RV antibodies (Arbitrary Units/mL)**						
Total		35.4 ± 2.7	37.2 ± 3.1	30.4 ± 1.6	35.1 ± 3.3	35.8 ± 3.4
IgM		5.7 ± 0.6	7.4 ± 0.8	7.6 ± 0.4	5.8 ± 0.4	7.0 ± 0.5
**Immunoglobulins (**μ**g/mL)**						
IgM		19.5 ± 0.8	19.1 ± 1.3	21.0 ± 0.8	19.2 ± 0.9	20.7 ± 2.5
IgG		3693.3 ± 180.2	5293.6 ± 695.7^*^	3851.9 ± 98.0	3779.9 ± 207.8	3295.5 ± 410.0
	IgG1	273.6 ± 6.7	191.6 ± 13.2^*^	133.3 ± 6.8^*^^#^	194.1 ± 8.7^*^	147.1 ± 12.7^*^^#^
	IgG2a	722.8 ± 17.4	672.2 ± 22.4	664.6 ± 51.2	722.9 ± 42.3	565.9 ± 53.9
	IgG2b	1055.8 ± 26.2	3185.4 ± 758.3^*^	1458.1 ± 50.6^*^^#^	1567.3 ± 123.4^*^^#^	1298.7 ± 267.2^#^
	IgG2c	1641.0 ± 170.4	1244.4 ± 63.8	1595.9 ± 40.6^#^	1295.6 ± 110.9	1283.9 ± 81.9
	Th1/Th2^a^	2.7 ± 0.2	5.1 ± 1.0^*^	3.8 ± 0.3^*^	3.1 ± 0.2	3.6 ± 0.1^*^
IgA		63.3 ± 1.5	91.4 ± 7.6^*^	73.9 ± 1.9^*^	72.7 ± 2.8^*^^#^	58.4 ± 6.3^#^

*Results are expressed as mean ± S.E.M. (n = 5–9)*.

* *p < 0.05 compared to REF (by MWU test)*.

# *p < 0.05 compared to RV (by MWU test)*.

a *Th1/Th2 ratio refers to the relationship between IgG2b+IgG2c and IgG1+IgG2a*.

The overall immunoglobulin production was modified in terms of IgG and IgA but not IgM (Table 3). Specifically, the RV group increased the total IgG levels, reflected in a threefold increase in IgG2b (Th1-associated isotype) compared to the REF group, and a slight decrease of IgG1 (Th2-associated isotype), which therefore increased the Th1/Th2 immunoglobulin ratio (*p* < 0.05). Moreover, the main protective immunoglobulin in mucosal sites, IgA, was also increased due to the infection (*p* < 0.05). The supplementation with the oligosaccharides partially prevented the changes in the production of IgG and IgA. On the one hand, animals receiving the oligosaccharides did not increase the total levels of IgG, although a slight increase in IgG2b was detected with respect to the REF group. Moreover, IgG1 levels were also lower, and this to a major extent for the supplementations with GOS/FOS, in which an increased Th1/Th2 ratio was seen (*p* < 0.05). On the other hand, the IgA production was increased in the RV+GOS/FOS and RV+2′-FL groups but not with the combination, the levels of which were similar to those of the REF group. Finally, *pIgR* and *Iga* gene expression in the small intestine was not affected either by RV infection or by the supplementations (data not shown). All these results suggest that the supplementations induce a pattern of immunoglobulins more similar to that of the REF group, probably indicating that a milder humoral response is required because other oligosaccharide-dependent mechanisms of protection are involved.

### Cytokine production

Some changes in a collection of cytokines present in the gut wash both at day 8 and day 16 were observed (Table 4). At day 8, corresponding to the peak of diarrhea, the concentration of all cytokines increased in the RV group (*p* < 0.05 for IL-6, IL-10, and IL-12), while the supplementation with GOS/FOS, 2′-FL or their combination prevented such increases. At the end of the infection (day 16), both RV and RV+2′-FL groups displayed a similar concentration of cytokines as the REF group. However, the RV+GOS/FOS group displayed less IL-1β and IL-10 than the RV group and the RV+GOS/FOS+2′-FL group showed lower concentrations for all studied cytokines with the exception of IL-4 (*p* < 0.05).

**Table 4:** Analysis of the concentration of intestinal cytokines in the gut wash during the peak of diarrhea (day 8) and at the end of the study (day 16).

		**REF**	**RV**	**RV+GOS/FOS**	**RV+2′-FL**	**RV+GOS/FOS+2′-FL**
**IL-1**β						
	d8	232.5 ± 37.3	376.3 ± 69.0	254.8 ± 32.7	204.6 ± 35.1^#^	220.7 ± 62.7
	d16	624.0 ± 116.9	542.0 ± 136.1	307.35 ± 70.8^*^	515.3 ± 99.6	298.6 ± 84.08^*^
**IL-6**						
	d8	375.6 ± 84.0	708.7 ± 149.5^*^	473.4 ± 75.7	344.8 ± 80.9^#^	394.5 ± 127.1
	d16	1439.2 ± 293.4	1137.24 ± 268.3	718.7 ± 182.7	1045.0 ± 178.77	686.6 ± 210.2^*^
**IL-4**						
	d8	28.3 ± 5.7	51.8 ± 9.6	34.9 ± 4.9	26.9 ± 5.71	31.0 ± 9.2
	d16	88.7 ± 17.7	75.3 ± 16.0	47.2 ± 12.0	67.6 ± 11.1	43.9 ± 12.9
**IL-10**						
	d8	916.0 ± 125.2	1407.3 ± 209.7^*^	951.9 ± 100.3	799.9 ± 145.1^#^	865.1 ± 223.2
	d16	1900.5 ± 288.1	1611.2 ± 315.2	965.4 ± 183.9^*^	1424.1 ± 191.3	919.2 ± 211.8^*^
**IL-12**						
	d8	1453.4 ± 184.3	2321.5 ± 289.2^*^	1470.2 ± 115.3	1385.2 ± 232.2^#^	1434.8 ± 285.9
	d16	2773.9 ± 520.8	2464.5 ± 407.8	1394.9 ± 381.6	2359.0 ± 377.3	1363.4 ± 315.9^*^^#^
**IFN-**γ						
	d8	579.0 ± 118.3	1097.6 ± 213.3	715.3 ± 100.8	541.0 ± 122.3^#^	603.0 ± 188.6
	d16	1972.1 ± 385.9	1589.4 ± 383.2	960.5 ± 231.3	1403.7 ± 225.0	918.1 ± 259.5^*^
**TNF-**α						
	d8	145.0 ± 27.5	251.2 ± 45.5	172.0 ± 25.2	133.4 ± 26.6^#^	149.9 ± 44.2
	d16	418.6 ± 81.1	363.6 ± 86.2	213.3 ± 52.2	314.8 ± 52.4	200.8 ± 56.3^*^

*Results are expressed as mean ± S.E.M. (n = 6)*.

* *p < 0.05 compared to REF (by MWU test)*.

# *p < 0.05 compared to RV (by MWU test)*.

**Figure 5:**
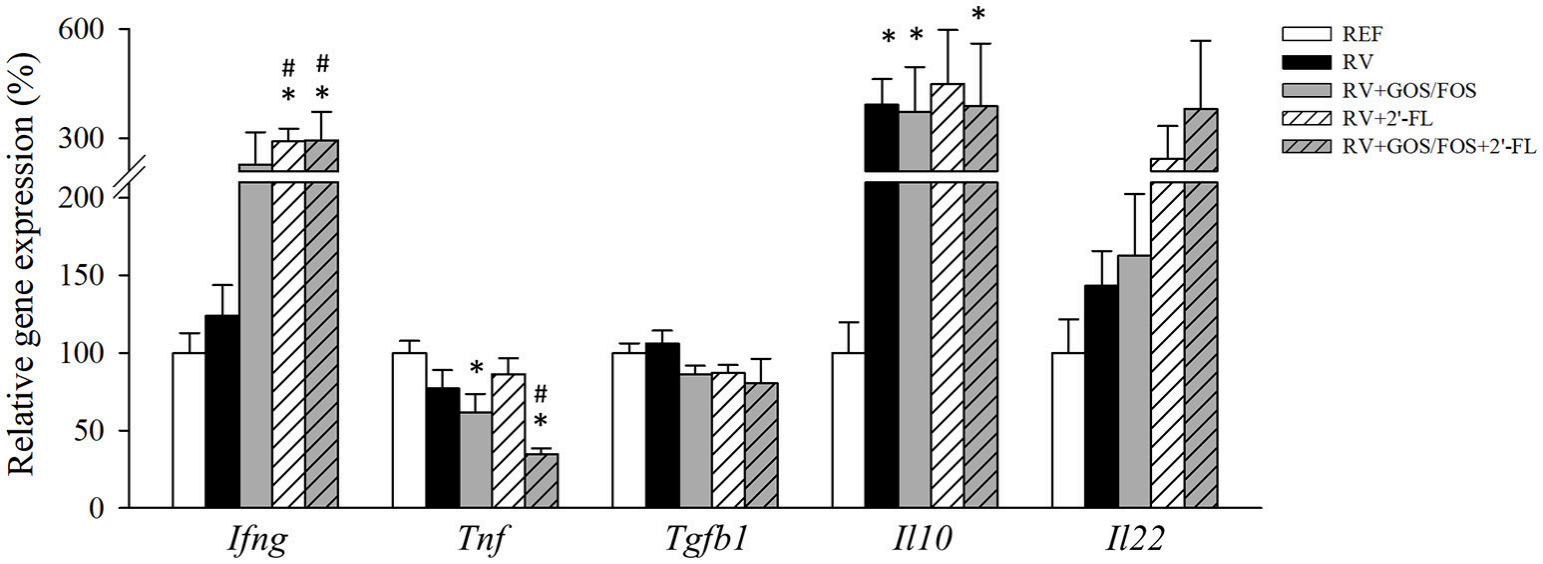
Assessment of cytokine gene expression in the small intestine. Cytokines were quantified in the gut wash by real-time PCR at the peak of diarrhea (day 8). Relative gene expression was calculated in respect to REF, which corresponded to 100% of transcription. Results are expressed as mean ± S.E.M. (*n* = 4–8). *p < 0.05 compared to REF group; ^#^*p* < 0.05 compared to RV group (by MWU test).

With regard to the intestinal gene expression of cytokines (Figure 5), the RV infection did not modify the expression of the antiviral cytokine IFN-γ, while the supplementations containing 2′-FL boosted its RNA levels, suggesting a possible role in the inhibition of the RV dissemination (Bass, 1997). *Tnf* gene expression was lower in both GOS/FOS groups with respect to the REF group (*p* < 0.05). The infection with RV tripled the expression of *Il10*, as seen in all RV-infected groups. *Tgfb1* and *Il22* gene expression was similar in all groups and *Il17* was not detected.

### Cecal SCFA production

**Figure 6:**
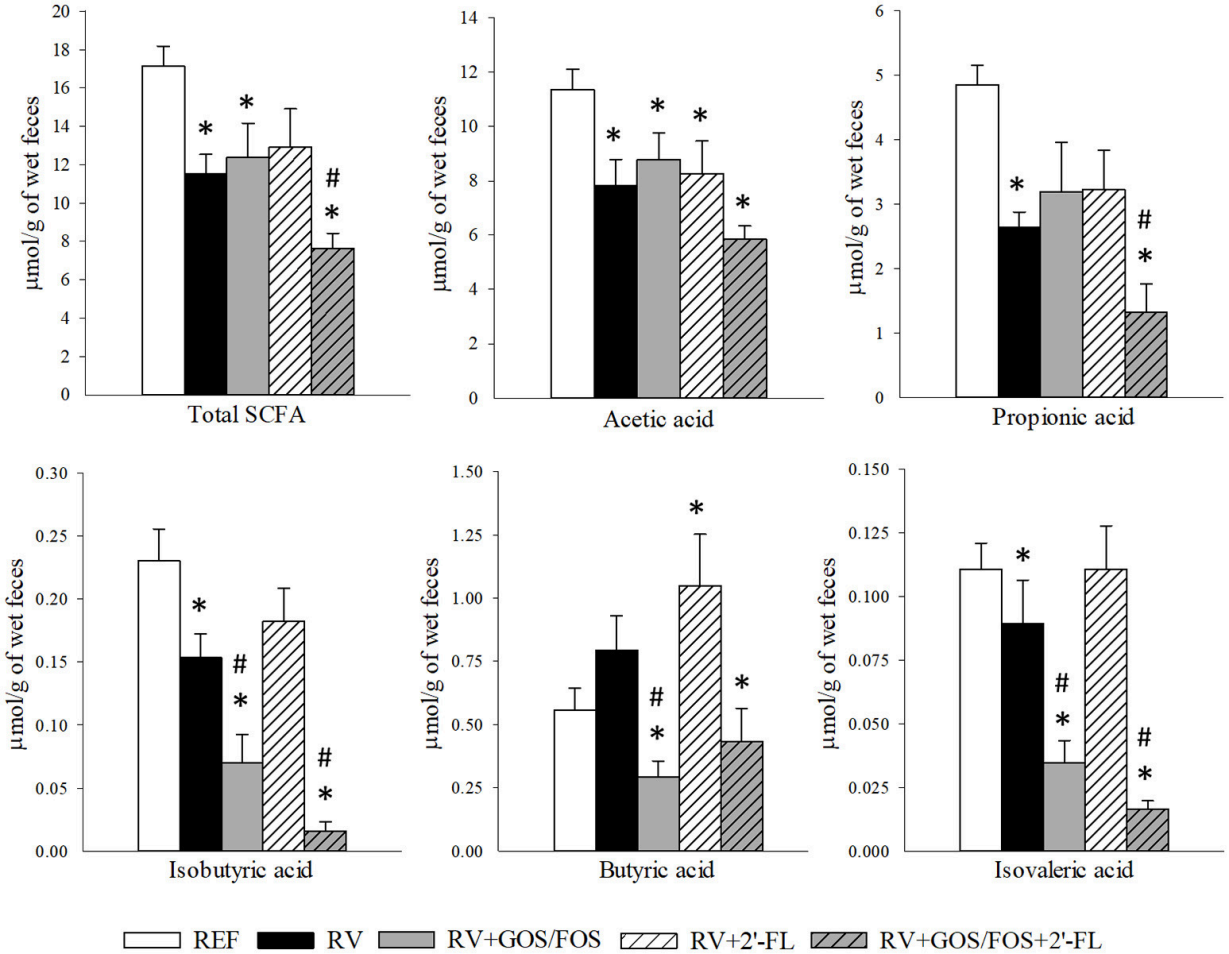
Cecal SCFA composition at the end of the study (day 16). Total SCFA, acetic, propionic, isobutyric, butyric and isovaleric acid production was quantified by HS-GC-MS. Results are expressed as mean ± S.E.M. (*n* = 9). *p < 0.05 compared to REF group; ^#^*p* < 0.05 compared to RV group (by MWU test).

The main metabolites of oligosaccharide breakdown by microbes are SCFA, which have well-known health-promoting effects on the host (Vandenplas et al., 2014; Corzo et al., 2015). The amount of total SCFA and acetic, propionic, isobutyric, butyric, isovaleric, valeric, caproic, and heptanoic acids produced in the cecum of the suckling rats were quantified at the latest time point of the study, thus allowing the observation of the effects after the 15-day long oligosaccharide supplementation (Figure 6). The amount of valeric, caproic and heptanoic acids found were below the limit of detection. The RV infection reduced the total cecal SCFA production with a consistent reduction of all SCFA, with the exception of butyric acid, which was not modified compared to REF. However, the supplementation with 2′-FL only reduced the amount of acetic acid (*p* < 0.05), the levels of all the others were similar to those of the REF group or even higher, as in the case of butyric acid (*p* < 0.05). With regard to the GOS/FOS supplementation alone, although it did not modify the total SCFA or the main SCFA, acetic and propionic acids with respect to the RV levels, it reduced the proportion of less abundant acids, such as isobutyric, butyric and isovaleric. Finally, the combination of GOS/FOS and 2′-FL showed differential effects to the supplementation of GOS/FOS alone, e.g., decreasing the total SCFA and propionic acid concentrations in the case of the RV group (*p* < 0.05).

## Discussion

Human milk fed infants seem to have a lower incidence and severity of infectious diseases compared to those receiving infant formula (Stuebe, 2009; Lamberti et al., 2011). Several bioactive compounds present in human milk are responsible for the protection against infections affecting mainly the gastrointestinal tract, such as the acute gastroenteritis produced in children primarily by rotavirus (RV). In particular, human milk oligosaccharides (HMOs) seem to be involved in the interception of pathogen attachment and to enhance the innate immune defense, among others (Bode, 2015). Herein, we have demonstrated for the first time that a purified 2′-FL intervention in a neonatal rat model of RV acute gastroenteritis reduces the associated clinical symptoms (i.e., it lowers the incidence, severity and duration) and that such an effect is related to the 2′-FL direct ability to promote intestinal maturation and to enhance neonatal immune responses. Other studies that exist assess the effect of HMO mixtures during RV infection, and therefore it is difficult to discern the effects of individual oligosaccharides and their specific mechanisms of action (Li et al., 2014; Comstock et al., 2017). However, it has to be taken into account that, although 2′-FL is the main oligosaccharide in human milk it is present but not so abundant in rat milk, which is dominated mainly by 3′-sialyllactose (3′-SL).

Moreover, it is the first time to assess the effects of the combination of 2′-FL and GOS/FOS, which kept those outcomes found separately for each of the products. Finally, we confirmed the preventive effect of GOS/FOS in RV diarrhea and its RV-blocking effect found in previous studies (Rigo-Adrover et al., 2017a,b) and also bring new data regarding its activity (e.g., beneficial histomorphometric changes and immunomodulatory effects).

In the present work, the RV SA11 induced a moderate diarrhea at some timepoint in nearly 90% of the infected animals. The non-supplemented and RV-administered animals influenced the neonatal immune response by increasing IgG systemic antibody levels and modulating the cytokine production during the peak of infection, as similarly observed in other studies (Fromantin et al., 1998; Azevedo et al., 2006). Furthermore, the infection caused alterations in the cecal SCFA profile.

Few studies have addressed the *in vivo* effects of HMOs on RV infection (Hester et al., 2013; Li et al., 2014). We have shown that the supplementation of 2′-FL given to neonatal rats before and during RV infection induced a clear protective role, significantly reducing the severity score, the incidence and duration of the diarrhea and the fecal weight. Because of the intrinsic effect on fecal consistency induced by GOS/FOS, as reported in previous studies (Rigo-Adrover et al., 2017b), its protective effect was only clearly observed after the normalization of data.

In the present study, the day of maximum viral shedding in the RV group was just 1 day post-RV inoculation, as already described in this model (Pérez-Cano et al., 2007; Rigo-Adrover et al., 2017a). Viral shedding was reduced up to 6 times by the GOS/FOS intervention, most probably due to its ability to bind to the virus, as it has been previously described as a mechanism of protection during the infective process (Rigo-Adrover et al., 2017a). The quantification of the virions by PCR would allow to confirm this hypothesis, as the quantification of RV gene copies would not be affected due to the binding with the product. In contrast, the intervention with 2′-FL did not reduce the viral shedding, despite a slight blocking of RV was seen in the *in vitro* assay, suggesting that virus binding would not be the main mechanism of action involved, at least for this RV strain, in ameliorating the diarrhea. In fact an *in vitro* study of Laucirica et al. (2017) found that 2′-FL may reduce infectivity, but in high doses. The ability of the HMOs to inhibit binding has been described in some experimental approaches studying different enteropathogens (Ruiz-Palacios et al., 2003; Etzold and Bode, 2014; Koromyslova et al., 2015; Weichert et al., 2016). These host–microbe–HMOs interactions can be explained by the similarities between the glycan motifs found in mammalian cells and in these types of oligosaccharides, which can truly act as decoy receptors (Donovan and Comstock, 2016).

Some RV diarrhea animal models, usually using the most virulent strains, induce serious intestinal malabsorption followed by fluid loss and dehydration, which ultimately affects body weight loss (Ciarlet et al., 2002; Jacobi et al., 2013). However, the RV SA11 strain induced a moderate diarrhea without weight loss. In this regard, GOS/FOS, but not 2′-FL intervention, promoted body weight gain at the end of the study regardless of the infective process. There is not a clear conclusion about the effects of the products on animal growth, as some studies find differences (Mezoff et al., 2016; Charbonneau et al., 2017) and some others not (Lima et al., 2017; Rasmussen et al., 2017; Rigo-Adrover et al., 2017b). Moreover, 2′-FL did promote the growth of the small intestine, which had a bigger percentage of the total body weight after 7 days of supplementation. This intestinal trophic effect of 2′-FL, which was even more evidenced and persistent in the GOS/FOS dietary intervention, has also been observed before for other prebiotic compounds, increasing the weight and promoting positive changes in the intestinal structure (Macfarlane, 2009; Rasmussen et al., 2017).

In our study, we did not detect major histological differences in the intestine during the peak of infection due to the RV. In fact, the absence of histopathological lesions in this model has also been reported previously (Guerin-Danan et al., 1998). Moreover, the lack of a clear barrier disruption could be linked to the mild diarrhea obtained in this particular model and even to the RV infective process that could be acting as a maturative challenge itself (Arévalo Sureda et al., 2017). Independently of the RV effects, GOS/FOS, but not 2′-FL, increased the villi height, villi area and the perimeter of the small intestine, which is in accordance with the observed gut growth-promoting effect. Several studies support this finding, as it has been shown that mice fed with 1% GOS diet prevented the villus height decrease induced by deoxynivalenol in the proximal small intestine (Akbari et al., 2017). Nevertheless, another study using a FOS diet did not observe any effect on villus height in growing rats (Daly and Shirazi-beechey, 2006). Regarding supplementation with HMOs, it is still unclear whether it affects intestinal architecture, since some studies found increased villi height in mice (Mezoff et al., 2016), others found a reduction after 2′-FL supplementation in preterm piglets (Rasmussen et al., 2017).

The present oligosaccharide interventions seem to be involved in driving the intestinal cell maturation. In order to establish the impact of both the RV inoculation and the nutritional intervention on the intestinal maturation process, the gene expression of two feasible markers of intestinal maturation in the neonatal rat were assessed during the peak of infection (Arévalo Sureda et al., 2017). On the one hand, it is well known that the neonatal constant fragment receptor (FcRn) mediates the binding and transfer of IgG across the small intestine during the suckling period of rats, whereas its expression is markedly decreased at weaning. On the other hand, although the expression of B-lymphocyte-induced maturation-protein-1 (Blimp-1) is present throughout all stages of rat life, it seems that it could act as a nuclear transcription repressor in the distal small intestine of enterocytes during the suckling period and lower its expression upon weaning (Arévalo Sureda et al., 2017). In our study, RV inoculation tended to reduce the gene expression of FcRn during the peak of infection, suggesting that probably RV challenge has a direct effect on intestine maturation. However, after interventional supplementations some significant differences were observed regarding FcRn but not Blimp-1. Animals receiving 2′-FL showed a trend toward lower levels of FcRn compared to the RV group, while the GOS/FOS supplementation reduced them significantly, suggesting that both seem to accelerate the intestinal development process in neonatal rats.

Another possible mechanism involved in the diarrhea amelioration could be an improvement in the epithelial barrier function conferring higher resistance to the infection. Herein, the higher levels of A1AT found in the RV group gut wash revealed an RV-induced disruption of the barrier integrity at the peak of infection. Such an effect was remarkably counteracted by 2′-FL and partially so in animals supplemented with GOS/FOS. Despite that, this enhanced barrier effect was not associated with changes in TJ protein gene expression. However, it is in line with a study showing that although GOS prevented monolayer integrity impairment and an acceleration of the TJ assembly *in vitro*, it did not affect the mRNA expression of CLDN4, OCLN, ZO1, and ZO2 (Akbari et al., 2017).

Intestinal mucins play an important role in the innate defense by limiting and neutralizing invading pathogens such as rotavirus (Kim and Khan, 2013). In the present study, the RV-stimulated MUC2 production in the small intestine was probably due to an attempt to control the infection. Such a response was prevented in animals supplemented with 2′-FL or GOS/FOS, which displayed mucin levels similar to those present in REF animals. Other authors have studied the impact of these types of prebiotics on mucin production and, for example, a FOS diet during 12 weeks decreased gene expression of MUC4 (Lima et al., 2017), while GOS affected goblet cell function (Bhatia et al., 2015). Per contra, a 4% HMOs blend supplemented in preterm pigs did not affect the intestinal gene expression of mucins 1 and 2 (Rasmussen et al., 2017).

Besides the action of 2′-FL and GOS/FOS on the maturing epithelial barrier, their effect on the developing immunity of the suckling rats is also of importance. Although some studies have already demonstrated the immunomodulatory action of GOS/FOS 9:1 (Rigo-Adrover et al., 2017a,b; Vonk et al., 2017), very few studies have analyzed the immune-related outcomes of nutritional interventions testing HMOs at clinical and preclinical levels. Moreover, most of these studies are mainly focused on growth and toxicology (Donovan and Comstock, 2016).

Anti-RV Ig has been found to be a good marker of infection and protection in previous studies in older animals (Pérez-Cano et al., 2007, 2008; Rigo-Adrover et al., 2017b). Here we have demonstrated that there is not a clear specific anti-RV Ig response due to SA11 infection, probably because of the immature immune system in early life. Thus, the effect of 2′-FL and GOS/FOS on the anti-RV immune response could not be properly assessed here. The total and anti-RV Ab found in all animals of the study may suggest a transference from their dams through breast milk. Furthermore, Li et al. (2014) did not find a modulation of HMOs in terms of total anti-RV IgG in a piglet model of infection. However, the global analysis of humoral immunity has shed some light regarding the changes in immune response due to the infection as well as due to the prebiotic interventions. Once the infection was resolved (day 16), the RV group showed higher levels of IgG, involving mainly an increase in an isotype associated with the Th1 response (IgG2b) and a decrease in that associated with the Th2 (IgG1). 2′-FL, GOS/FOS and their combination were able to counteract the IgG2b increase due to RV by reducing the levels of total IgG to reference values. These results may indicate that the oligosaccharides supplementation avoided the active immune response against the virus, likely due to the exclusion mechanisms mentioned before (i.e., blocking effect and enhancing barrier integrity).

Moreover, to determine the effects of RV and supplementations on the cellular response, the intestinal levels of cytokine gene expression at the peak of infection (day 8) and their production on both day 8 and day 16 (post-diarrhea period) were assessed. The RV infection produced significant increases in both pro-inflammatory/Th1 (IL-12, IL-6) and anti-inflammatory/Th2 (IL-10) cytokines at the peak of infection, as found in several rotavirus infection models (Gandhi et al., 2017). The higher intestinal IL-10 content is in accordance with the higher gene expression in the intestinal tissue of infected animals found herein and also in other models of RV infection (Li et al., 2014). This increase, together with that found for MUC2, appears to be directed by the host with the aim of counteracting the RV aggression toward the intestinal tissue. In the present study, 2′-FL, but not GOS/FOS, was able not only to control much of the cytokine production boosted by the RV but also to decrease the secretion of other proinflammatory cytokines (IL-1β, IL-6, IL-12, IFN-γ, TNFα), suggesting its high immunomodulatory role. In fact, it is suggested that HMOs, such as 2′-FL, can mimic selectin ligands and directly bind to immune cells and trigger signaling pathways that can ultimately affect immune cell populations and cytokine secretion (Donovan and Comstock, 2016). Specifically, it has been shown that 2′-FL was able to induce PBMC IL-10 and IFNγ production *in vitro* under stimulatory conditions (Donovan and Comstock, 2016); ileocecal-resected mice fed 2′-FL, upregulated distal small intestine immune response genes (Mezoff et al., 2016); and finally, infants fed with infant formula with 2′-FL had similar cytokine levels to breastfed infants, which are lower than those of infant formula-fed infants (Goehring et al., 2016).

Finally, the effect of RV infection and oligosaccharide supplement on cecal SCFA was established. Intestinal SCFA production after prebiotic fermentation decreases pH and inhibits potentially pathogenic bacteria (Gibson et al., 2005). Moreover, it is known that SCFA may affect epigenetic gene regulation in the intestinal mucosa by a change in the histone acetylation promoted by butyric acid (Sanderson, 2004). Our results indicated that the reduction in SCFA due to RV infection was not prevented by the oligosaccharide supplementations, although some enhancing effect of 2′-FL on butyric acid was found. The lack of effect on this variable is in line with other studies in which 12 weeks' supplementation of FOS *in vivo* did not affect acetic and propionic production (Lima et al., 2017). Some other approaches were negative in finding SCFA differences due to HMO mixtures or 2′-FL (Donovan and Comstock, 2016; Rasmussen et al., 2017). Moreover, the higher proportion of butyric acid found in our study after 2′-FL supplementation could be linked to the enhanced barrier integrity (Kau et al., 2012).

One of the objectives of this study was to ascertain whether the combination of both prebiotic compounds may induce more beneficial effects than those given separately. Although both prebiotics have similar effects on several variables (i.e., diarrhea reduction and immune response modulation), each compound also has differential effects (i.e., GOS/FOS has the highest blocking effect whereas 2′-FL is the strongest barrier promoter). The combination of both oligosaccharides presented all the activities found in each prebiotic separately and even had an additive effect in some variables. Thus, the combination is as effective as the supplementation alone regarding some variables, such as the intestinal trophic effect, the SA11 viral shedding modulation and the butyric production. However, several features only attained significant differences or appeared to be more evident with the combination of GOS/FOS and 2′-FL. Thus, a clear additive effect is found on the trophic effect observed on the large intestine on day 8, inducing lower AUC for severity and incidence, potentiating the *in vitro* blocking activity against the virus, counteracting the RV-induced increases in certain Ig isotypes (IgG2b and IgA), or promoting higher intestinal maturation by means of inducing lower FcRn gene expression, among others.

In summary, the RV infection in neonatal rats induces a diarrhea process that involves the alteration of the immune response. The preventive daily supplementation of a scGOS/lcFOS 9:1 prebiotic mixture, 2′-FL (the main HMO found in human milk), or a combination of both is able to partially prevent the RV-induced diarrhea in this preclinical model. The mechanisms involved in such protective action included specific effects on the two lines of defense: immunomodulation and enhancement of the intestinal barrier function. An additive effect of the combination of GOS/FOS with 2′-FL was also found in several mechanisms. These results indicate that these compounds in combination could be helpful when added to infant formulas, to protect against human RV-induced diarrhea in children.

## Data availability statement

The datasets generated and/or analyzed during the current study are available from the corresponding author on reasonable request.

## Author contributions

IA-B, MM-C, KK, BvL, BS, JG, MR-L, ÀF, MC, FP-C were involved in the design and/or execution of the experiments. IA-B and FP-C analyzed and interpreted the results and drafted the paper. All the authors contributed to the critical revision of the manuscript. All authors read and approved the final version of the manuscript for publication.

### Conflict of interest statement

The authors declare that they have a financial relationship with the organization that sponsored the research. KK, BvL, BS, and JG are employees of Nutricia Research. JG is head of the Division of Pharmacology, Utrecht Institute for Pharmaceutical Sciences, Faculty of Science at the Utrecht University and partly employed by Nutricia Research. BvL as indicated by the affiliations, is leading a strategic alliance between University Medical Centre Utrecht/Wilhelmina Children's Hospital and Nutricia Research. The remaining authors declare that the research was conducted in the absence of any commercial or financial relationships that could be construed as a potential conflict of interest.
